# Estimation of clonal diversity in HTLV-1 infection

**DOI:** 10.1186/1742-4690-11-S1-O50

**Published:** 2014-01-07

**Authors:** Daniel J Laydon, Anat Melamed, Aaron Sim, Nicolas A Gillet, Kathleen Sim, Sam Darko, J Simon Kroll, Daniel C Douek, David A Price, Charles RM Bangham, Becca Asquith

**Affiliations:** 1 Section of Immunology, Wright-Fleming Institute, Imperial College School of Medicine, London, UK; 2 Centre for Integrative Systems Biology and Bioinformatics, South Kensington Campus, Imperial College, London, UK; 3 Department of Molecular and Cellular Epigenetics, University of Liège, Liège, Belgium; 4 Section of Paediatrics, Wright-Fleming Institute, Imperial College School of Medicine, London, UK; 5 Vaccine Research Center, National Institutes of Health, Bethesda, USA; 6 Institute of Infection and Immunity, Cardiff University School of Medicine, Cardiff, Wales, UK

## 

Within hosts, Human T-Lymphotropic Virus Type-1 (HTLV-1) is spread through de novo infection and infected cell proliferation, producing multiple T cell clones (infected cells with the same genomic proviral integration site). Between hosts, the number of clones observed from a 10μg sample of DNA varies by up to three orders of magnitude. The question arises: what is the total number of clones in the host from which that sample was drawn? Considering each clone as a “species”, the question becomes analogous to the “unseen species problem” in population ecology. We tested four species richness (number of species) estimators, and a novel approach, “*DivE*”, using three independent datasets: (i) viral populations from patients infected with HTLV-1, (ii) T cell antigen receptor clonotype repertoires, and (iii) microbial data from infant faecal samples. In all datasets, *DivE* was substantially more accurate than the ecological estimators, which were strongly biased by sample size when applied to datasets where the majority of species was not already present. *DivE* can also be used to estimate with accuracy the population clone structure from small samples. Previous estimates of HTLV-1 clone diversity in vivo were in the order of 102, and have increased in line with method sensitivity. In contrast, the mean estimated number of clones in the circulation of a single host (asymptomatic carriers and patients with chronic inflammation) by *DivE* was more than two logs higher than previously estimated. These estimates will inform our understanding of the dynamics and pathogenesis of HTLV-1 infection.

